# Calpain-Mediated Degradation of Drebrin by Excitotoxicity *In vitro* and *In vivo*

**DOI:** 10.1371/journal.pone.0125119

**Published:** 2015-04-23

**Authors:** Takahiko Chimura, Thomas Launey, Nobuaki Yoshida

**Affiliations:** 1 Laboratory of Developmental Genetics, Center for Experimental Medicine and Systems Biology, Institute of Medical Science, The University of Tokyo, Tokyo, Japan; 2 RIKEN Brain Science Institute, Launey Research Unit, Wako, Saitama, Japan; Univ. Kentucky, UNITED STATES

## Abstract

The level of drebrin, an evolutionarily conserved f-actin-binding protein that regulates synaptic structure and function, is reduced in the brains of patients with chronic neurodegenerative diseases such as Alzheimer’s disease (AD) and Down’s syndrome (DS). It was suggested that excitotoxic neuronal death caused by overactivation of NMDA-type glutamate receptors (NMDARs) occurs in AD and DS; however, the relationship between excitotoxicity and drebrin loss is unknown. Here, we show that drebrin is a novel target of calpain-mediated proteolysis under excitotoxic conditions induced by the overactivation of NMDARs. In cultured rodent neurons, degradation of drebrin was confirmed by the detection of proteolytic fragments, as well as a reduction in the amount of full-length drebrin. Notably, the NMDA-induced degradation of drebrin in mature neurons occurred concomitantly with a loss of f-actin. Furthermore, pharmacological inhibition of f-actin loss facilitated the drebrin degradation, suggesting a functional linkage between f-actin and drebrin degradation. Biochemical analyses using purified drebrin and calpain revealed that calpain degraded drebrin directly *in vitro*. Furthermore, cerebral ischemia also induced the degradation of drebrin *in vivo*. These findings suggest that calpain-mediated degradation of drebrin is a fundamental pathology of neurodegenerative diseases mediated by excitotoxicity, regardless of whether they are acute or chronic. Drebrin regulates the synaptic clustering of NMDARs; therefore, degradation of drebrin under excitotoxic conditions may modulate NMDAR-mediated signal transductions, including pro-survival signaling. Overall, the results presented here provide novel insights into the molecular basis of cellular responses to excitotoxicity *in vitro* and *in vivo*.

## Introduction

Alzheimer’s disease (AD) and vascular dementia are the most prevalent types of dementia among the elderly [1], and the term “vascular cognitive impairment” (VCI) was introduced to describe the heterogeneous group of cognitive disorders that share a presumed vascular cause [2]. Vascular risk factors such as hypertension, smoking, diabetes, and hypercholesterolemia are common to both VCI and AD [2,3]. Elderly adults with dementia often show evidence of both AD (such as neuritic plaques and neuronal fibrillary tangles) and VCI (such as cerebral or lacunar infarctions) [1], suggesting potential synergy in the appearance of symptoms and even convergence of the underlying cellular and molecular pathways.

Glutamate is the major excitatory neurotransmitter in the mammalian central nervous system. N-Methyl-D-aspartate receptors (NMDARs) are glutamate-gated calcium channels that play important roles in memory and learning. Overactivation of these receptors is toxic to cultured neurons [4,5] as well as brain tissues *in vivo* [6,7], and is thought to contribute to neuronal loss in both acute neurodegenerative diseases such as stroke [4–10] and chronic neurodegenerative diseases such as AD [11,12]. Memantine, an open-channel blocker that preferentially inhibits overactivated NMDARs, shows significant positive effects on the cognition of patients with moderate to severe AD [13]. In addition, several NMDAR antagonists can protect neurons from ischemic damage in animal models [14–18]. These findings indicate that excitotoxicity caused by overactivation of NMDARs plays a central role in the pathogenesis of chronic and acute neurodegenerative diseases. To understand the pathogenesis and refine the therapeutic strategies for these diseases, it is crucial to elucidate the cellular responses to overactivation of NMDARs and the molecular basis of the resulting neuronal death.

Administration of a lethal or sublethal dose of NMDA to cultured neurons activates a variety of signaling molecules, including neural nitric oxide synthase [19], phosphatidylinositol-4,5-bisphosphate 3-kinase [20], extracellular signal-related kinase 1/2 [21,22], calcineurin [23], and caspases and calpains [24–26]. In addition to neuronal death, this type of NMDA treatment also elicits morphological changes in dendritic spines and the loss of actin fibers (f-actin). Several proteins are also degraded under these conditions; for example, calpains degrade the cytoskeletal protein spectrin [27], synaptic proteins such as the NR2A and NR2B subunits of NMDARs, and postsynaptic density protein 95, a major scaffolding protein that anchors signaling molecules at the postsynaptic membrane [28–30]. These findings suggest that proteins that regulate cytoskeletal and synaptic functions are regulated quantitatively during the course of NMDA-mediated excitotoxicity; hence, identification and characterization of proteolytic substrates is critical to unveil the molecular mechanisms involved in excitotoxicity.

Drebrin is an evolutionarily conserved actin-binding protein in the brain [31–34]. The embryonic-type isoform (drebrin E) and the adult-type isoform (drebrin A) are produced by alternative splicing. The isoform conversion from drebrin E to drebrin A is promoted in parallel with the maturation of neurons [35]. Whereas drebrin E is distributed in the soma of a variety of cell types, drebrin A is expressed specifically in neurons and localizes preferentially to the dendritic spines of mature neurons [36–38]. Overexpression of drebrin A in primary cortical or hippocampal neurons elongates dendritic spines and enhances spine motility [39,40], whereas down-regulation of drebrin A in primary hippocampal neurons decreases the density and width of dendritic spines and inhibits synaptic clustering of NMDARs [40]. Furthermore, drebrin A knockout mice show defects in context-dependent freezing after fear conditioning [41], which may be related to the pivotal role that drebrin A plays in the regulation of synaptic transmission by altering actin polymerization in dendritic spines. Furthermore, neuronal levels of drebrin are reduced in patients with AD [42], Down’s syndrome (DS) [43], and even mild cognitive impairment [44], which is a putative prodromal stage of AD [45], suggesting that drebrin may serve as an important molecular indicator of brain pathophysiology. However, the molecular mechanisms underlying the decreased expression of drebrin in pathophysiological conditions remain poorly understood.

Here, we show that NMDA-induced excitotoxicity elicits the degradation of drebrin in primary hippocampal and cortical neurons within several hours, and that this process is triggered by calcium influx and mediated by calpains. Furthermore, a functional link between f-actin and the degradation of drebrin was identified. Experimental cerebral ischemia, an *in vivo* model of brain injury caused by excitotoxicity [4,18], also induced the degradation of drebrin. Overall, the results presented here indicate that excitotoxicity induces calpain-mediated degradation of drebrin *in vitro* and *in vivo*, and suggest that this mechanism may be a cause of drebrin loss in chronic neurodegenerative diseases such as AD and DS.

## Results

### NMDA-induced excitotoxicity promotes the degradation of drebrin

**Fig 1:**
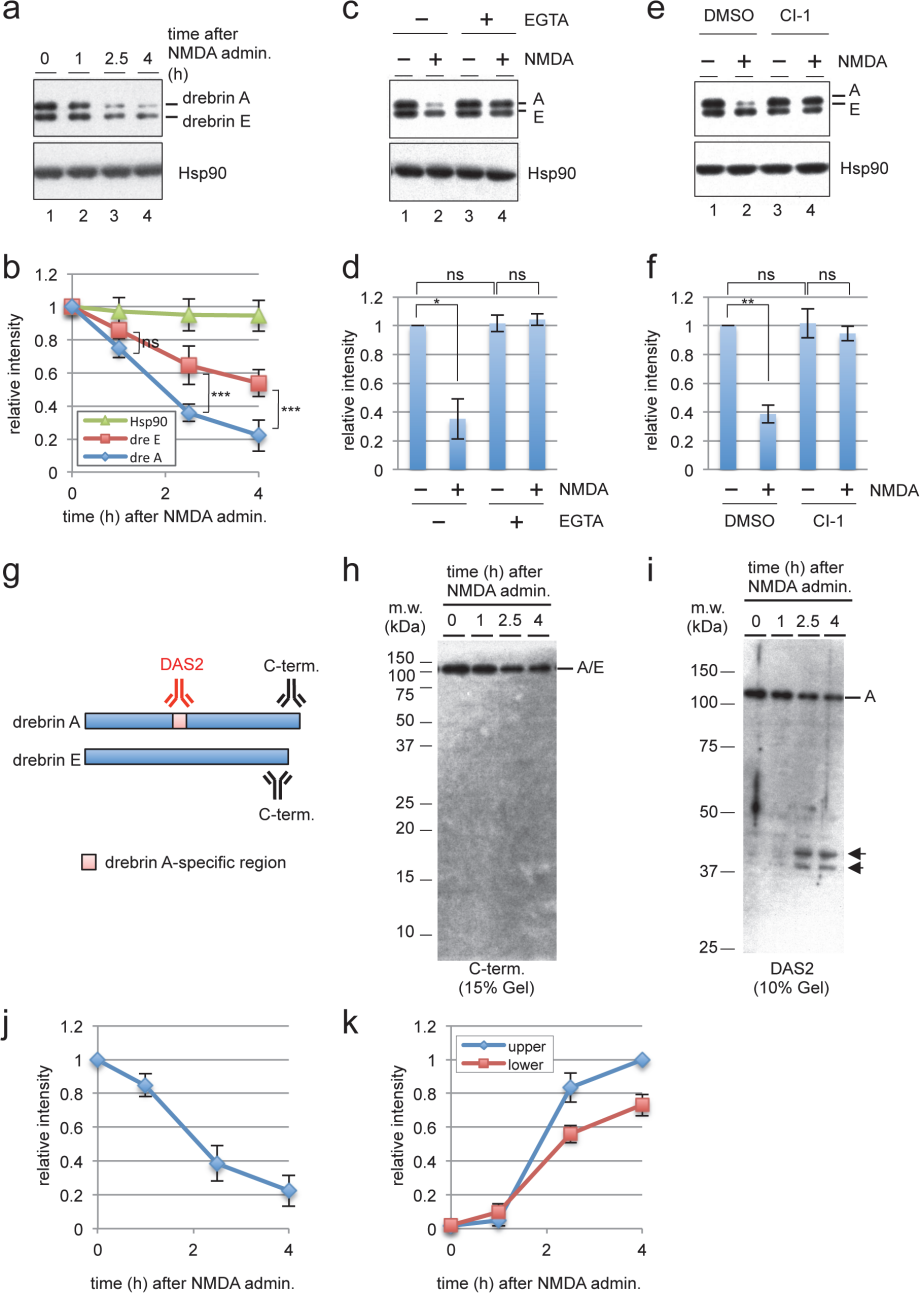
NMDA-mediated excitotoxicity induces the degradation of drebrin in cultured rat hippocampal neurons. (a) Western blot analyses of drebrin A (dre A) and drebrin E (dre E) in untreated and NMDA-treated primary rat hippocampal neurons. The expression level of Hsp90 was used as a loading control. (b) Quantification of the signal intensities of drebrin A, drebrin E, and Hsp90 shown in (a), relative to those at time 0 h. The data are represented as the mean ± standard deviation of n = 4 replicates. ***P < 0.005 by a Student’s t-test; ns, not significant. (c, e) Western blot analyses of drebrin A and drebrin E in untreated and NMDA-treated primary rat hippocampal neurons that were pretreated with EGTA for 30 min (c) or calpain inhibitor-I (CI-1) for 1 h (e). (d,f) Quantification of the signal intensities of drebrin A shown in (c) and (e), relative to those in the control samples (EGTA (-)/NMDA (-) for (c) and DMSO/NMDA(-) for (e)). The data are represented as the mean ± standard deviation of n = 3 replicates. *P < 0.05 and **P < 0.01 by a Student’s t-test; ns, not significant. (g) Schematic illustration of the antibodies used in this study. The C-term antibody recognized drebrin A and drebrin E, and the DAS2 antibody recognized drebrin A only because the epitope was located in a drebrin A-specific region. (h, i) Western blot analyses of extracts of NMDA-treated neurons using the C-term (h) and DAS2 (i) antibodies. The two major degradation products detected by the DAS2 antibody are indicated by arrows. (j) Quantification of the signal intensities of full-length drebrin A in (i) relative to those at time 0 h. (k) Quantification of the signal intensities of the two degradation products in (i) relative to that of the upper band at 4 h after NMDA treatment. The data are represented as the mean ± standard deviation of n = 3 replicates.

To determine the effect of NMDA-induced excitotoxicity on the stability of drebrin, primary rat hippocampal neurons were treated with NMDA for several hours, and the amount of drebrin protein was analyzed by western blotting (Fig 1A). The maturation of neurons was confirmed by the localization of drebrin in dendritic spines (S1 Fig). Exposure of the cells to 30 μM NMDA induced apparent neuronal death after 24 h (S2 Fig); therefore, this concentration was used as an excitotoxic dose. Two high and low molecular weight bands representing drebrin isoforms A and E, respectively, were detected in untreated cells (Fig 1A, lane 1). After the administration of NMDA, the drebrin A signal decreased but the drebrin E signal was relatively stable (Fig 1B), suggesting that drebrin A is preferentially degraded in response to excitotoxicity. Since drebrin A is enriched in mature neurons, we assume that the response to NMDA occurs preferentially in mature neurons.

Because NMDAR is a calcium channel, we examined whether influx of extracellular calcium is required for the NMDA-induced degradation of drebrin A. Prior to the administration of NMDA, the cultured cells were treated with 2.5 mM ethylene glycol tetraacetic acid (EGTA) for 30 min to chelate extracellular calcium. As shown in Fig 1C and 1D, 2.5 h after NMDA administration, the degradation of drebrin A was suppressed by the addition of EGTA, indicating that NMDA-induced influx of extracellular calcium triggers the degradation of drebrin A.

Calpains are calcium-dependent cysteine proteases; therefore, we examined whether inhibition of these enzymes suppresses NMDA-induced degradation of drebrin. Pretreatment of neurons with 10 μM calpain inhibitor-I suppressed the degradation of drebrin A caused by exposure to NMDA (Fig 1E and 1F). These results indicate that calpains are required for the excitotoxic degradation of drebrin A. Calpains catalyze limited cleavage of target proteins; therefore, if drebrin A is processed by calpains directly, degradation products with lower molecular weights should be produced. To test this possibility, extracts of NMDA-treated neurons were separated in gels containing a high concentration of polyacrylamide, and western blot analyses were performed using an antibody recognizing the C-terminal region of drebrin A and drebrin E (used in Fig 1A–1E and referred to as “C-term” in Fig 1G), and an antibody recognizing a drebrin A-specific region [36] (DAS2; Fig 1G). No degradation products were detected using the C-term antibody (Fig 1H); however, two bands of approximately 40 kDa were detected using the DAS2 antibody (Fig 1I–1K), suggesting that calpain degrades drebrin A directly. Notably, these 40 kDa fragments were not produced when the cells were pretreated with EGTA or calpain inhibitor-I (Parts A and B of S3 Fig, respectively). In addition, no degradation products were detected by the M2F6 antibody, which was specific to both drebrin A and drebrin E [46] (S4 Fig), suggesting that the epitope regions recognized by the M2F6 [47] and C-term antibodies contain one or more cleavage sites and are inactivated by proteolysis, or that the neighboring regions of the epitopes contain multiple cleavage sites.

### The relationships between drebrin and f-actin

**Fig 2:**
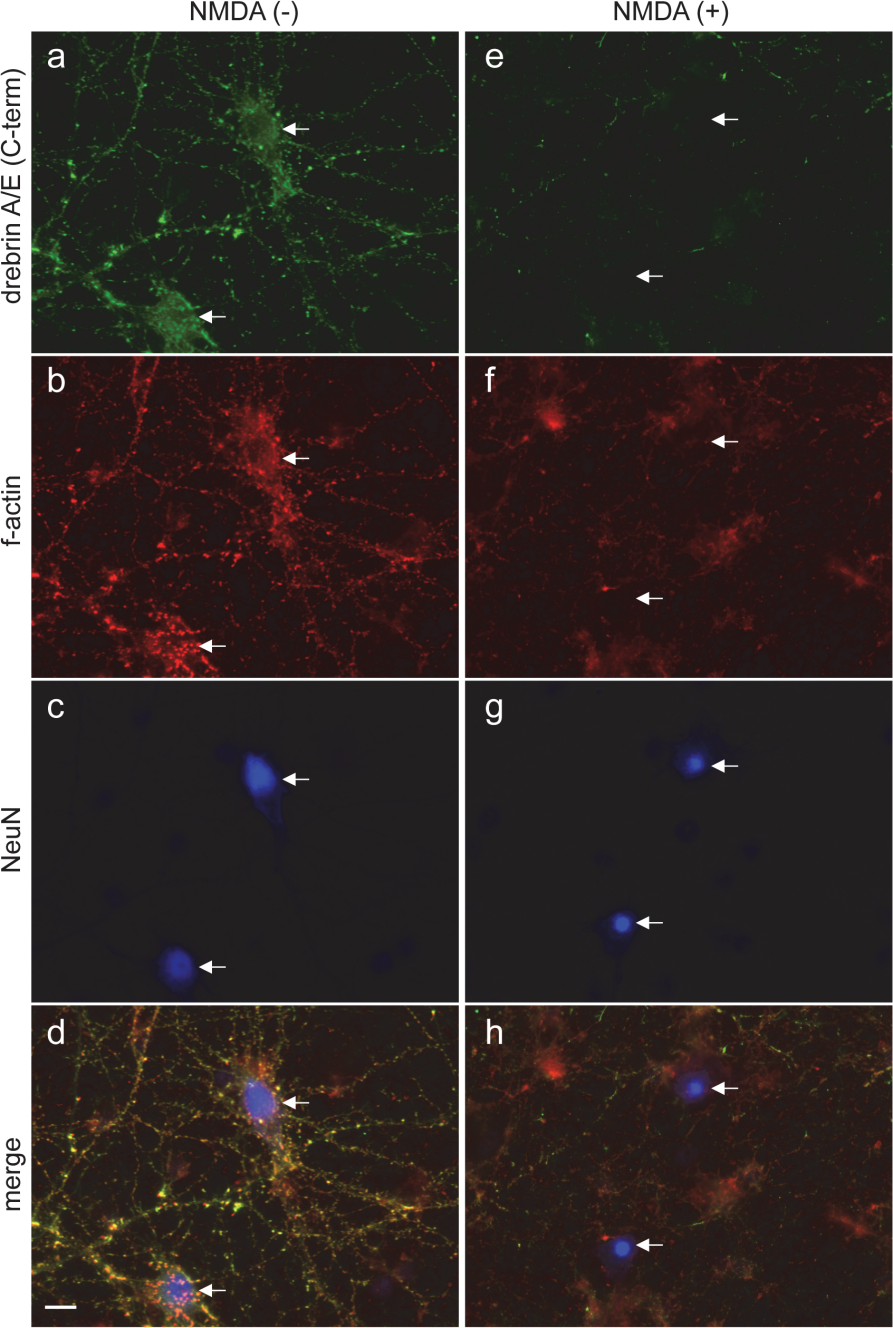
Losses of f-actin and drebrin occur concomitantly after NMDA treatment. (a–h) Immunostaining of rat hippocampal neurons with antibodies against drebrin A/E (C-term), NeuN, and phalloidin (f-actin). (a–d) Control neurons. (e–h) Neurons that were treated with NMDA for 4 h. The arrows indicate NeuN-positive cells (neurons). Scale bar: 10 μm.

Drebrin is an f-actin-binding protein [34,48]; therefore, degradation of drebrin likely affects the state of actin polymerization. To test this possibility, immunocytochemistry was used to detect drebrin and f-actin expression in NeuN-positive cells (mature neurons) in rat primary hippocampal cultures. In control cultures without NMDA (Fig 2A–2D), drebrin A/E was colocalized with f-actin in NeuN-positive cells, as reported previously [48]. Consistent with the western blot analyses (Fig 1), exposure of the cultures to NMDA apparently reduced the expression of drebrin A/E in NeuN-positive neurons (Fig 2E–2H). Furthermore, f-actin expression was also reduced in NMDA-treated NeuN-positive neurons. Quantitative analyses of the intensities of the drebrin and f-actin signals in 100 randomly selected NeuN-positive neurons revealed that the reduction in drebrin expression occurred concomitantly with the reduction in f-actin levels in most of the NMDA-treated neurons (S5 Fig).

**Fig 3:**
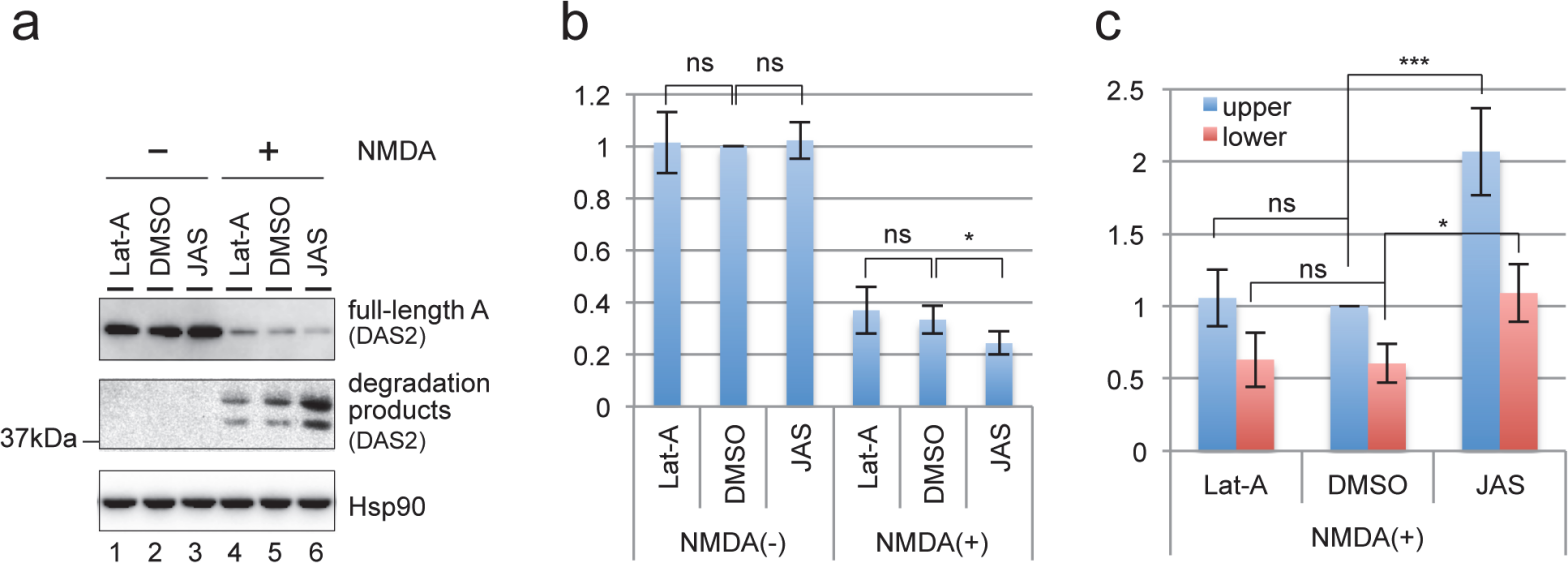
Functional relationship between f-actin and the degradation of drebrin. (a) The effects of stabilization of f-actin using 5μM JAS and destabilization of f-actin using 2μM Lat-A on the levels of NMDA-induced proteolytic fragments of drebrin A in rat hippocampal neurons. The expression level of Hsp90 was used as a loading control. (b) Quantification of the signal intensities of full-length drebrin A shown in (a), relative to those in the control sample (DMSO/NMDA(-)). (c) Quantification of the signal intensities of the two degradation products in (a), relative to that of the upper band in the DMSO/NMDA(+) sample. The data are represented as the mean ± standard deviation of n = 4 replicates. *P < 0.05 and ***P < 0.005 by a Student’s t-test; ns, not significant.

Next, the stability of f-actin was modulated using Latrunculin-A (Lat-A), an f-actin-destabilizing agent, and jasplakinolide (JAS), an f-actin-stabilizing agent. Rat hippocampal cultures were pretreated with these agents for 1 h and then treated with NMDA to induce the degradation of drebrin A. Destabilization of f-actin by Lat-A did not affect the efficiency of NMDA-induced degradation of drebrin A (Fig 3A–3C). In addition, treatment of the neurons with Lat-A alone, to cause forced loss of f-actin, did not induce the degradation of drebrin A (Fig 3A, lane 1), indicating that drebrin A degradation is not a direct consequence of f-actin loss. Exposure of the neurons to JAS increased the amount of degradation products induced by NMDA treatment (Fig 3A, compare lanes 5 and 6; and Fig 3C). Accordingly, a slight but statistically significant enhancement of the NMDA-induced decrease in full-length drebrin A expression was also observed in JAS-treated neurons (Fig 3A and 3B), suggesting that stabilized f-actin facilitates the degradation of drebrin A.

### Drebrin A is a direct target of calpain *in vitro*

**Fig 4:**
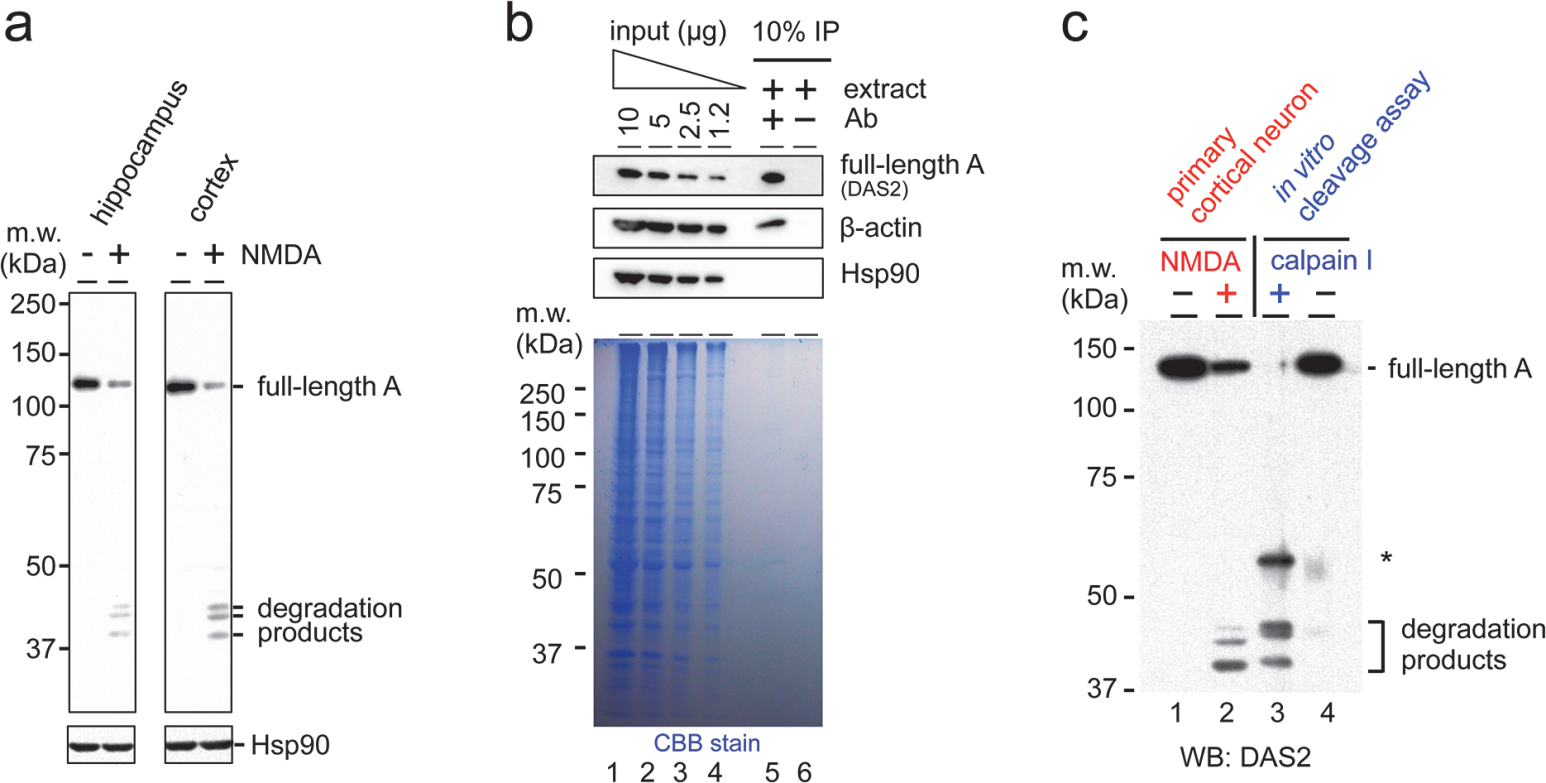
Calpain degrades drebrin A directly *in vitro*. (a) Western blot analyses of drebrin A degradation products in mouse hippocampal and cortical neurons treated with NMDA. Western blotting was performed using the DAS2 antibody. The expression level of Hsp90 was used as a loading control. (b) Purification of mouse drebrin A by IP. Western blotting was performed using the DAS2 antibody, anti-β-actin antibody, and anti-Hsp90 antibody (upper panels) to detect the amount of these proteins in the input and IP fractions. Coomassie Brilliant Blue (CBB) staining was used to detect the total amount of protein in each fraction. (c) An *in vitro* cleavage assay in which purified drebrin A was incubated with or without calpain. The asterisk indicates a degradation product that was detected specifically in the *in vitro* cleavage assay. Western blotting was performed using the DAS2 antibody. The experiment was repeated three times with similar results.

To determine whether the observed NMDA-induced degradation of drebrin A occurs in multiple species, the experiments were repeated in mouse primary hippocampal and cortical neuronal cultures. The maturation of neurons was confirmed by the localization of drebrin in dendritic spines (S6 Fig). Treatment of these neurons with NMDA for 2.5 h induced the degradation of drebrin A (Fig 4A) and three major degradation products of approximately 40 kDa were detected.

Next, an *in vitro* cleavage assay was performed to determine whether calpain degrades drebrin A directly. Drebrin A was purified from protein extracts of mouse cerebral cortex by immunoprecipitation (IP) using the C-term antibody. The amount of drebrin A in the input and IP fractions was measured by western blotting (Fig 4B, upper panel), and the total amount of protein in each fraction was visualized by Coomassie Brilliant Blue staining (Fig 4B, lower panel). Approximately equivalent amounts of drebrin A were detected in 10 μg of the input fraction (Fig 4B, lane 1) and 10% of the IP fraction (Fig 4B, lane 5). On the other hand, Coomassie Brilliant Blue staining showed that most of the proteins detected in 10 μg of the input fraction (lane 1) were absent from the IP fraction, confirming that the IP procedure successfully purified drebrin A. The observation that β-actin, a known binding partner of drebrin [31], but not Hsp90 was co-purified with drebrin also verified the specificity of the IP procedure and indicated that purified drebrin A maintains its tertiary structures and activities *in vivo*. Immunoprecipitated drebrin A bound to the C-term antibody, and protein G-sepharose was resuspended in cleavage buffer and incubated with or without purified calpain (Fig 4C, lane 3 and 4, respectively). Incubation with calpain reduced the amount of full-length drebrin A and produced several degradation products (Fig 4C, lane 3), indicating that calpain cleaves drebrin A directly *in vitro*. Notably, three fragments of approximately 40 kDa were detected in both the *in vitro* cleavage assay and primary cortical neurons treated with NMDA, suggesting that calpain also degrades drebrin A directly in cultured neurons. However, the molecular weights of the degradation products produced in the neuronal cultures and *in vitro* differed slightly. To confirm whether this phenomena is commonly observed *in vitro*, or specifically occurs when purified drebrin was used, the cleavage assay was performed using crude cortical extract as substrates (S7 Fig). In this condition, the molecular weights of degradation products detected in *in vitro* cleavage assay were almost the same as those in cultured neurons (S7 Fig). It is possible that the tertiary structures of drebrin in the presence of drebrin antibody might affect cleavage sites by calpain [49]. Alternatively, the SDS-PAGE migration of proteins in purified samples might be slower than in crude samples as observed in the case of β-actin (Fig 4B).

### Drebrin A is degraded in response to ischemia *in vivo*

**Fig 5:**
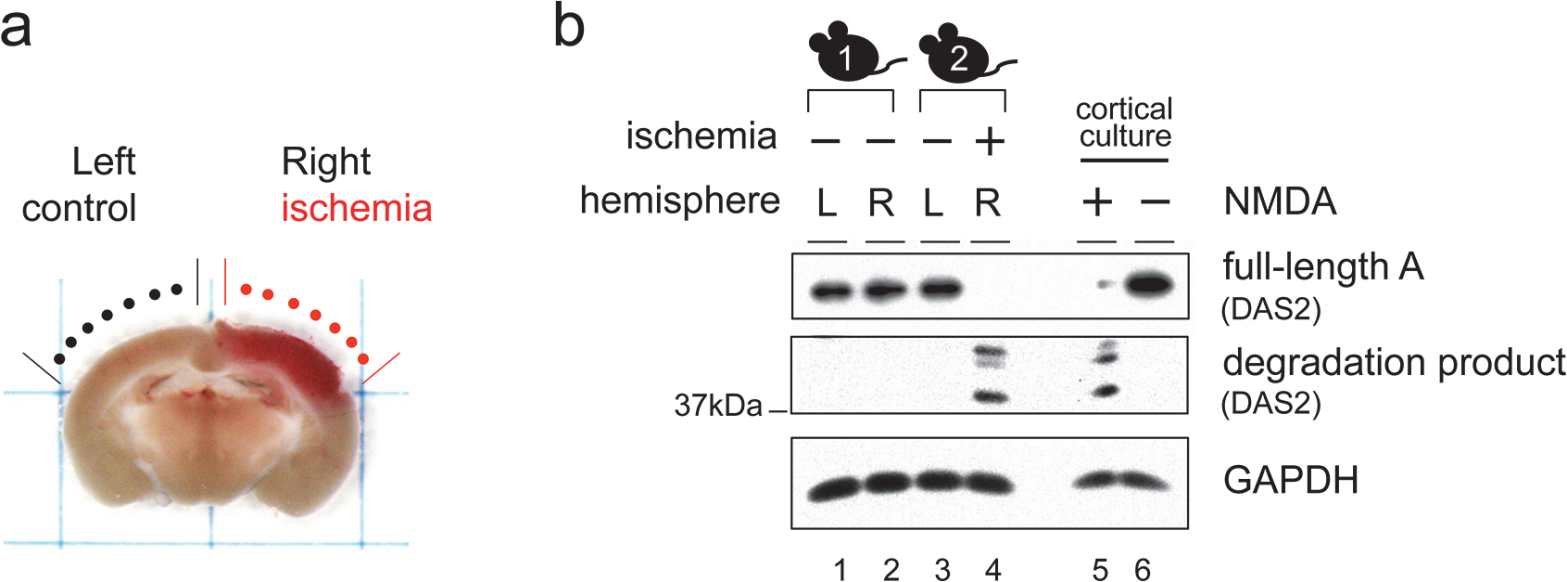
Ischemic brain damage induces the degradation of drebrin A. (a) Image of a mouse coronal brain slice showing the site of induction of photothrombotic ischemia (red) at the right hemisphere. The left hemisphere was used as a control. (b) Western blot analyses of drebrin A in total protein extracts of the left hemisphere (control) and the ischemic right hemisphere of the mouse brain. The DAS2 antibody was used to detect full-length drebrin A and its degradation products. The expression level of GAPDH was used as a loading control. The experiment was repeated three times with similar results.

To determine whether drebrin is degraded under excitotoxic conditions *in vivo*, ischemic brain damage was specifically induced in the right hemisphere of the mouse cerebral cortex using photothrombosis. Briefly, the photosensitive dye rose bengal was activated by white light illumination to cause damage to blood vessels [50]. The ischemic locus could be identified without staining 3 h after photothrombosis (Fig 5A). Total proteins were extracted from the ischemic locus and the corresponding contralateral region (the left hemisphere), and degradation of drebrin A was examined by western blotting using the DAS2 antibody. Ischemic damage reduced the amount of full-length drebrin A and induced the formation of proteolytic fragments with the same molecular weight as those detected *in vitro* (Fig 5B, compare lanes 4 and 5). The amount of drebrin A in the contralateral region (Fig 5B, lane 3) was similar to that in the control mouse without photothrombosis (Fig 5B, lanes 1 and 2), suggesting that the degradation of drebrin A was induced in the ischemic region specifically. Overall, these results indicate that drebrin A is degraded under excitotoxic conditions both *in vivo* and *in vitro*.

## Discussion

### Drebrin is a novel target of proteolysis under excitotoxic conditions

**Fig 6:**
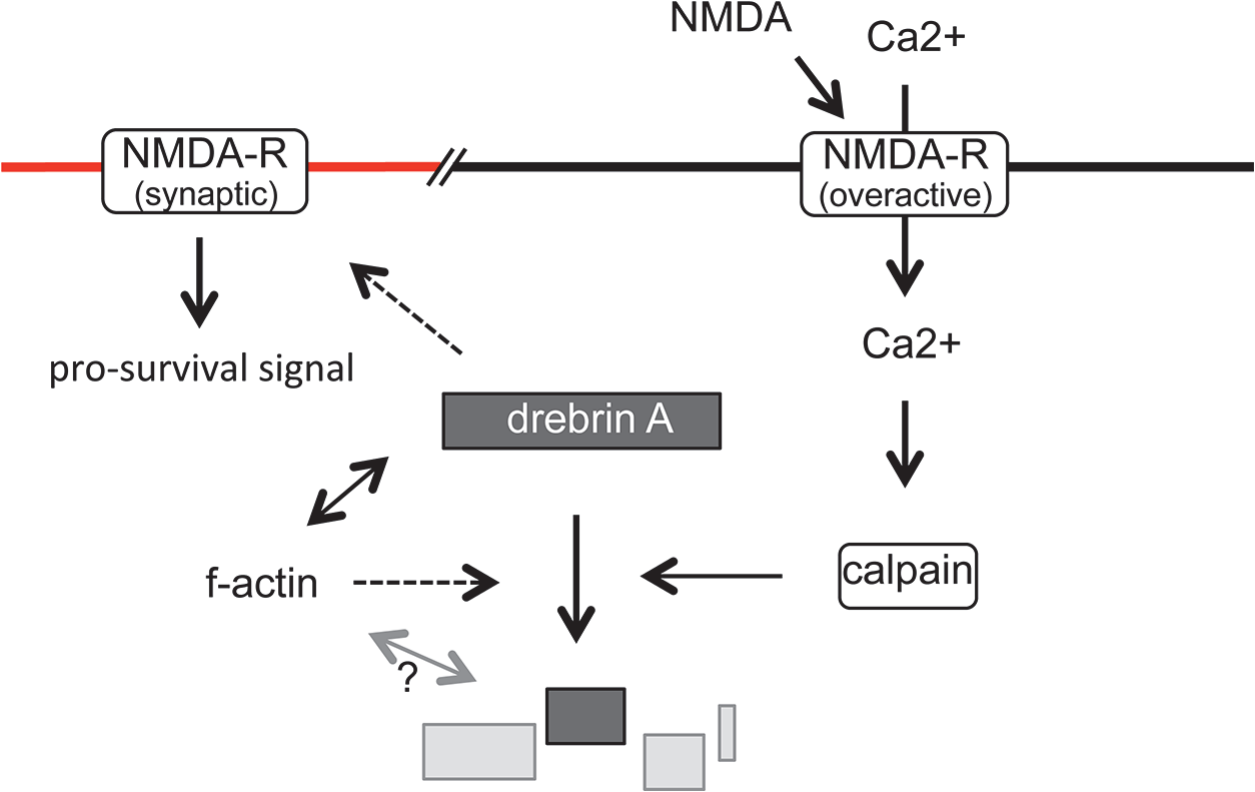
Schematic representation of the signaling cascades from excitotoxic stimulation to the degradation of drebrin. NMDA induces the degradation of drebrin A in a calpain-dependent manner. Because drebrin A plays a critical role in clustering synaptic NMDARs, which transduce pro-survival signals, degradation of drebrin might modulate these signals and result in the delay or acceleration of neuronal death. The solid arrows indicate direct effects, and the dashed arrows indicate that effects have not yet been determined as direct.

The results presented here indicate that drebrin is a novel target of proteolysis under excitotoxic conditions induced by NMDA, and that the degradation of drebrin depends on calcium influx and calpain activity (Fig 6). Calpain degraded purified drebrin A directly and produced several degradation products with molecular weights and proteolytic patterns that were similar to those in NMDA-treated cultured neurons (Fig 4) and ischemic brain sections (Fig 5), suggesting that drebrin A is a direct target of calpain both *in vitro* and *in vivo*. A bioinformatics search of drebrin A using the GPS-CCD package [51] did not uncover any obvious potential calpain cleavage sites. This result is complicated further by the anomalous migration of drebrin on SDS-PAGE gels. In future studies, the actual cleavage sites will need to be determined experimentally.

It is possible that the loss of activity of full-length drebrin plays a role in signaling during excitotoxicity. In addition to the loss of drebrin, exposure of NeuN-positive mature neurons to NMDA also caused a reduction in the amount of f-actin (Fig 2). Drebrin stabilizes f-actin *in vitro* [52] and in cells [53,54], and down-regulation of drebrin A in rat cultured neurons by antisense oligonucleotides suppresses the accumulation of f-actin in dendritic spines [55]; therefore, degradation of drebrin likely facilitates the loss of f-actin under excitotoxic conditions. Examining the efficiency of f-actin loss using a proteolysis-resistant mutant of drebrin A will be important in future studies. Notably, forced stabilization of f-actin by treatment of neurons with JAS facilitated the degradation of drebrin A (Fig 3A–3C), indicating that its degradation is modulated by the extent of f-actin loss (Fig 6). This process might be an alert system to detect and prevent insufficient f-actin loss under excitotoxic conditions. F-actin is recognized as a damage-associated molecular patterns (DAMPs) by the immune system [56,57]; therefore, the accelerated degradation of f-actin-stabilizing proteins such as drebrin in response to insufficient f-actin loss may prevent unwanted immune responses in neurological diseases *in vivo*. However, the molecular mechanisms underlying this process remain elusive. Given that forced f-actin loss by treatment of neurons with Lat-A had no effect on the efficiency of drebrin degradation (Fig 3A–3C), the physical interaction between drebrin and f-actin is unlikely to affect the susceptibility of drebrin to proteolysis. However, there might be an unidentified excitotoxicity-specific interaction between f-actin and drebrin that affects the efficiency of proteolysis. Alternatively, JAS treatment might enhance the effect of NMDA. This hypothesis is consistent with previous reports showing that JAS treatment or gene disruption of the actin-severing protein gelsolin, both of which result in the stabilization of f-actin, facilitate the elevation of intracellular calcium levels in neurons by glutamate [58,59]. It is an important issue in future studies to examine whether the facilitation of drebrin degradation is also observed in the neurons lacking actin-severing protein such as gelsolin [59].

So far, we have discussed the possible functional effect(s) of decreased amounts of full-length drebrin A. It is also possible that the degradation products of drebrin retain some of the activities of the full-length protein or acquire new activities. Notably, the epitopes recognized by the DAS2 antibody are located adjacent to the f-actin-binding region [54,60], suggesting that the degradation products possess f-actin-binding activity (Fig 6). Drebrin interacts physically not only with f-actin, but also with the postsynaptic scaffolding protein Cupidin/Homer2 [61], the gap junction protein connexin 43 [62], the microtubule binding protein EB3 [63], and the transcriptional co-activator Spikar [64]. Therefore, it is important to determine whether drebrin degradation products affect the activities of these proteins or other unidentified partners.

### Relationships between Drebrin and NMDAR activation

Brief and transient stimulation of neurons induces a rapid NMDAR-dependent translocation of drebrin from dendritic spines to dendrites within several minutes, without changes in its expression level [65,66]. In this case, re-entry of drebrin into dendritic spines occurs shortly after withdrawal of glutamate receptor stimulants [66] and the neurons remain intact even 24 h after the transient stimulation [65]. By contrast, here, prolonged activation of NMDARs induced neuronal death 24 h after the stimulation (S2 Fig), as well as the proteolytic degradation of drebrin within several hours. Several previous reports suggested that synaptic and extrasynaptic NMDARs play distinct roles in neuronal death; specifically, activation of synaptic NMDARs transduces pro-survival signal, whereas activation of extrasynaptic NMDARs induces neuronal death [22,67–69]. Neuronal activation by co-application of bicuculline, a GABA(A) receptor blocker, and 4-aminopyridine, a potassium channel blocker, both of which are used commonly to stimulate synaptic NMDARs [69,70] induces the rapid translocation of drebrin [66], suggesting that differences in the location of activated NMDARs causes differential outputs in terms of drebrin degradation and neuronal death.

Because drebrin A facilitates the synaptic clustering of NMDARs [40], proteolysis of drebrin A by calpain potentially modulates NMDAR-mediated pro-survival signals under excitotoxic conditions (Fig 6). Several proteins related to synaptic NMDAR signaling, such as the NR2A subunit of the NMDAR [29,30,71,72] and postsynaptic density protein 95, a synaptic scaffolding protein for NMDAR [28,30], are degraded by calpain in response to overactivation of NMDARs [73,74]. Together with the results presented here, these findings suggest that excitotoxic conditions induce the orchestrated degradation of components involved in the regulation of the functions of synaptic NMDARs, which can accelerate or delay neuronal death. Pharmaceutical agents that maintain or enhance synaptic NMDAR-mediated pro-survival signaling are potential therapeutic strategies for the effective treatment for AD and/or VCI patients not only during, but also after, the overactivation of NMDARs; hence, it will be important to determine the effect of degradation of proteins such as drebrin on synaptic NMDAR-mediated pro-survival signaling.

### Drebrin degradation *in vivo*

VCI and AD share a number of risk factors and can coexist in elderly adults with dementia [1–3]. Notably, amyloid beta oligomers induce the overactivation of NMDARs in cultured neurons [75–77] and render neuronal cells vulnerable to excitotoxicity [78]. On the other hand, ischemia in rat models [79] and NMDA treatment of primary neuronal cultures [80] induce the accumulation of amyloid beta protein at the peripheral zone of the infarct and around dead neurons, respectively. These results suggest that the production of amyloid beta protein and overactivation of the NMDAR comprise a positive feedback loop, and that even a small infarct can trigger the propagation of neuronal degeneration and amyloid beta accumulation; this feedback loop is a putative molecular mechanism underlying the close relationship between VCI and AD. The brains of patients with neurological diseases such as AD and DS have lower levels of drebrin than normal brains [42–44]. Because the neurodegeneration observed in DS patients shows AD pathology (i.e., the accumulation of amyloid beta protein) [81,82], the loss of drebrin in DS brains is quite reasonable. Here, ischemia induced the degradation of drebrin *in vivo*, suggesting that drebrin loss is a novel and common pathology of neurodegenerative diseases, regardless of whether they are chronic or acute. Calpain is activated in AD [83]; hence, it is likely that drebrin loss in AD and DS patients is caused by calpain-mediated proteolysis. Examining the presence of proteolytic fragments of drebrin A in the brains of AD/DS patients or animal models would address this possibility. In previous studies, the mouse monoclonal M2F6 antibody [46] was used for detection of full-length drebrin A/E in the brains of neurological patients [42–44] and AD model mice [84]; however, this antibody did not detect any proteolytic fragments of drebrin in our experiments (S4 Fig). Therefore, the use of the DAS2 antibody would be critical for this purpose.

In summary, this study demonstrates that excitotoxic conditions caused by the overactivation of NMDARs *in vitro* and ischemic brain injury *in vivo* induce the degradation of drebrin A. Biochemical analyses revealed that drebrin A is a direct target of calpain. Because drebrin loss occurs in chronic neurodegenerative diseases such as AD and DS [42–44], degradation of this protein may be a fundamental pathology of a wide variety of neurodegenerative diseases. In clinical settings, memantine is used to inhibit the overactivation of NMDARs and enhance neuronal survival. Drebrin plays an important role in the synaptic clustering of NMDARs [40]; hence, analyses of the relationship between the degradation of drebrin and synaptic NMDAR-mediated pro-survival signaling may aid the development of alternative therapeutic strategies for neurodegenerative diseases.

## Materials and Methods

### Chemicals

NMDA and calpain inhibitor-I were obtained from Sigma-Aldrich (St. Louis, MO, USA), EGTA was obtained from Wako Chemicals (Osaka, Japan), Jasplakinolide and Latrunculin A were obtained from Enzo Life Sciences (Farmingdale, NY, USA), and rose bengal was obtained from Nacalai Tesque (Kyoto, Japan).

### Antibodies

The anti-drebrin A/E (C-term, #MX823) antibody was obtained from Progen Biotechnik (Heidelberg, Germany), the anti-drebrin A/E (M2F6) antibody was obtained from Enzo Life Sciences, the anti-drebrin A (DAS2, #28023) antibody was obtained from IBL (Gunma, Japan), the anti-HSP90 (#sc-7947) and anti-β-actin (#sc-69879) antibodies were obtained from Santa Cruz Biotechnology (Santa Cruz, CA, USA), the anti-GAPDH (#MAB374) antibody was obtained from Merck Millipore (Darmstadt, Germany), and the anti-NeuN (#ab128886) antibody was obtained from Abcam (Cambridge, MA, USA).

### Animals and ethics statement

Mice were housed under pathogen-free conditions in the experimental animal facility at the University of Tokyo. Rats were sacrificed on the day of arrival. All surgery was performed under sodium pentobarbital anesthesia, and all efforts were made to minimize suffering. Experiments using rats and mice were approved by the Animal Care and Use Committee of the University of Tokyo (Approval Numbers PA10-60 and PA09-36, respectively). The animals were handled in strict accordance with the ARRIVE guidelines and the guidelines of the Animal Care and Use Committee of the University of Tokyo.

### Primary neuronal cultures

Hippocampal neurons were prepared from Sprague-Dawley rat embryos at embryonic day 18 (Nihon SLC Inc., Shizuoka, Japan). The cells were plated onto poly-L-lysine-coated 24-well plates at a density of 1.25 × 10^5^ cells/well in neuron culture medium (Neurobasal (Life Technologies, Tokyo, Japan) supplemented with B27 (Life Technologies) and L-glutamine (Life Technologies)). Cortical and hippocampal neurons were prepared from Jcl:MCH mouse embryos at embryonic day 16 (Japan CLEA, Tokyo, Japan). The cells were plated onto poly-L-lysine-coated 24-well plates at a density of 2 × 10^5^ cells/well in neuron culture medium.

### Induction of excitotoxicity *in vitro*

For rat neurons, cultures at 15–17 days *in vitro* were treated with 30 μM NMDA for 2.5 h, unless otherwise stated. For mouse neurons, cultures at 10–12 days *in vitro* were treated with 30 μM NMDA for 2.5 h. Pretreatments with calpain inhibitor-I or EGTA were performed 1 h or 30 min before the NMDA treatment, respectively.

### Immunocytochemistry

Rat hippocampal neurons were cultured on poly-L-lysine-coated coverslips. After treatment with NMDA for 4 h, the cells were fixed with 4% (w/v) paraformaldehyde/0.1 M sodium phosphate buffer (pH 7.1) for 1.5 h at 4°C, permeabilized with blocking buffer (0.1 M sodium phosphate buffer containing 0.4% (v/v) Triton X-100, 2% (v/v) donkey serum, and 1% (w/v) bovine serum albumin) for 30 min at room temperature, and then labeled with anti-drebrin A/E (C-term) and anti-NeuN antibodies at 4°C overnight. Alexa 488-conjugated donkey anti-mouse IgG (Invitrogen) and Alexa 647-conjugated donkey anti-rabbit IgG (Invitrogen) were used as secondary antibodies. F-actin was stained with Alexa 555-conjugated phalloidin (Invitrogen). For quantitative analyses of the immunofluorescence data, images of 100 randomly selected NeuN-positive neurons were collected using a BIOREVO BZ-9000 microscope (Keyence, Osaka, Japan) with a 100× oil immersion objective, and the signal intensities of the circular areas (40 μm in diameter) covering both the cell soma and the apical dendrites were measured using Image J analysis software.

### Purification of drebrin A by IP

The C-term antibody (5 μl; #MX823, Progen Biotechnik) and protein G-sepharose (5 μl; GE Healthcare Japan, Tokyo, Japan) were mixed with 200 μl of 1% PBST buffer (1× PBS containing 1% (v/v) Triton-X100 and protease inhibitor cocktail), rotated for 2 h at 4°C, and then washed three times with 1% PBST buffer. Subsequently, mouse brain extract (300 mg) was added to the antibody-beads complex, rotated overnight at 4°C, and washed three times with 1% PBST. The mouse brain extracts were prepared by homogenizing the cerebral cortexes of 2-month-old (17–19 g) female C57BL/6J mice (Nihon SLC Inc.) in 1% PBST.

### *In vitro* cleavage assay

Fifty micrograms of mouse cortical extract or purified drebrin A bound to the C-term antibody and protein G-sepharose were resuspended in 40 μl of cleavage buffer (40 mM HEPES-KOH, pH 7.3, 1 mM CaCl_2_, and 5 mM DTT) containing 20 ng of purified calpain-1 (Calbiochem) and incubated at 37°C for 10 min. The cleavage reaction was terminated by adding 40 μl of 2× Laemmli sample buffer, followed by boiling.

### Induction of ischemia

Photothrombotic ischemia was induced as described previously [50]. Briefly, 2-month-old (17–19 g) female C57BL/6J mice (Nihon SLC Inc.) were anesthetized and rose bengal photosensitive dye (15 mg/ml in sterile PBS, corresponding to 0.15 mg/g of body weight) was delivered intraperitoneally 5 min before photoactivation. A midline scalp incision was made to expose the skull. For illumination, a fiber-optic bundle of a cold light source (Luminar Ace-150TX; Hayashi Watch Works, Tokyo, Japan) with a 4 mm aperture was centered at 2 mm posterior and 2 mm right from the bregma. Two mice were used in each experiment, one of which was a “no-ischemia” control. Another four mice were used to confirm reproducibility.

### Protein extracts and western blot analyses

Proteins were extracted from primary cultures in 24-well plates using Laemmli sample buffer. For protein extracts from ischemic brains, the ischemic region and corresponding contralateral region were excised and homogenized in T-Buffer (120 mM NaCl, 1 mM EDTA, 20 mM Tris-HCl, pH 7.5, 0.5% (v/v) Triton X-100, and protease inhibitor cocktail). The lysates were centrifuged to remove the insoluble matter and Laemmli sample buffer was added to the supernatants. ECL Prime (GE Healthcare Japan) was used to detect the DAS2 antibody and Pierce ECL Plus (Thermo Scientific, Rockford, IL, USA) was used to detect all other antibodies. Densitometric analyses of bands were performed using Image J analysis software.

### Statistics

All statistical tests were performed using KyPlot 5.0 software (KyenceLab Inc., Tokyo, Japan).

## Supporting Information

S1 Fig Localization of drebrins at dendritic spines in the rat hippocampal neurons used in this study. Immunostaining of rat hippocampal neurons at 17 days *in vitro* (DIV) with antibody against drebrin A/E (M2F6) and phalloidin (f-actin). Scale bar: 10 μm.
(TIF)

S2 Fig Exposure to 30 μM NMDA induces death of rat primary hippocampal neurons. (a) Representative images of rat hippocampal neuronal cultures that were treated with NMDA for the indicated times and stained with trypan blue. Neurons were defined as cells with a large soma and extended dendrites. Scale bar: 50 μm. (b) Quantification of cell death measured by trypan blue staining. Approximately 300–500 cells were counted for each condition in each independent experiment. The numbers represent the percentages of trypan blue-positive cells. The asterisk indicates a statistically significant difference (*P* = 1.56 × 10^–6^ by a Tukey-Kramer test) compared with the non-treated (0 h) condition (ns, not significant). The data are represented as the mean ± standard deviation of n = 3 replicates.
(TIF)

S3 Fig EGTA and calpain inhibitor-I suppress the excitotoxicity-induced degradation of drebrin in cultured rat hippocampal neurons. (a) Western blot analyses of drebrin A proteolytic fragments in neurons that were pretreated with EGTA for 30 min (a) or calpain inhibitor-I for 1 h (b), and then exposed to NMDA. The samples used in Fig 1C and 1E were reanalyzed using the DAS2 antibody in (a) and (b), respectively. The experiments were repeated a minimum of three times with similar results.
(TIF)

S4 Fig The M2F6 antibody detects reduced amounts of full-length drebrin but no degradation products. (a) Detection of NMDA-induced decreases in the levels of drebrin A and E using the M2F6 antibody. The expression level of Hsp90 was used as a loading control. (b) Lack of signals derived from degradation products recognized by the M2F6 antibody.
(TIF)

S5 Fig Losses of f-actin and drebrin occur concomitantly after NMDA treatment. (a) The signal intensities (AU, arbitrary units) of drebrins A and E (drebrin A/E) and f-actin in circular areas (40 μm diameter) that covered the cell soma and proximal dendrites of 100 randomly selected NeuN-positive neurons. Each dot represents one neuron. The thresholds indicated by orange lines are set at 40 percent or 70 percent of median values of drebrin or f-actin in NMDA(-) condition, respectively. (b) Schematic representation showing the identification of four quadrants (Q1-Q4). (c) Statistical analysis of the numbers of neurons included in each quadrant (Q1-Q4). The data are represented as the mean ± standard deviation of n = 3 replicates. ***P < 0.005 by a Student’s t-test.
(TIF)

S6 Fig Localization of drebrins at dendritic spines in the mouse neurons used in this study. Immunostaining of mouse cortical (left panels) and hippocampal neurons (right panels) at 12 days *in vitro* (DIV) using antibodies against drebrin A/E (M2F6) and phalloidin (f-actin). Scale bar: 10 μm.
(TIF)

S7 Fig Calpain degrades drebrin A directly *in vitro*. An *in vitro* cleavage assay using crude brain cortical extract as a substrate in the absence or presence of 100μM calpain inhibitor-1 (CI-1). The asterisks indicate the degradation products detected specifically in the *in vitro* cleavage assay. The arrow indicates a non-specific band.
(TIF)
