# Supplementary figures and images for: Calpain-Mediated Degradation of Drebrin by Excitotoxicity In vitro and In vivo
Source: PLoS One. 2015 Apr 23;10(4):e0125119. doi: 10.1371/journal.pone.0125119 (PMC4408054; doi:10.1371/journal.pone.0125119)

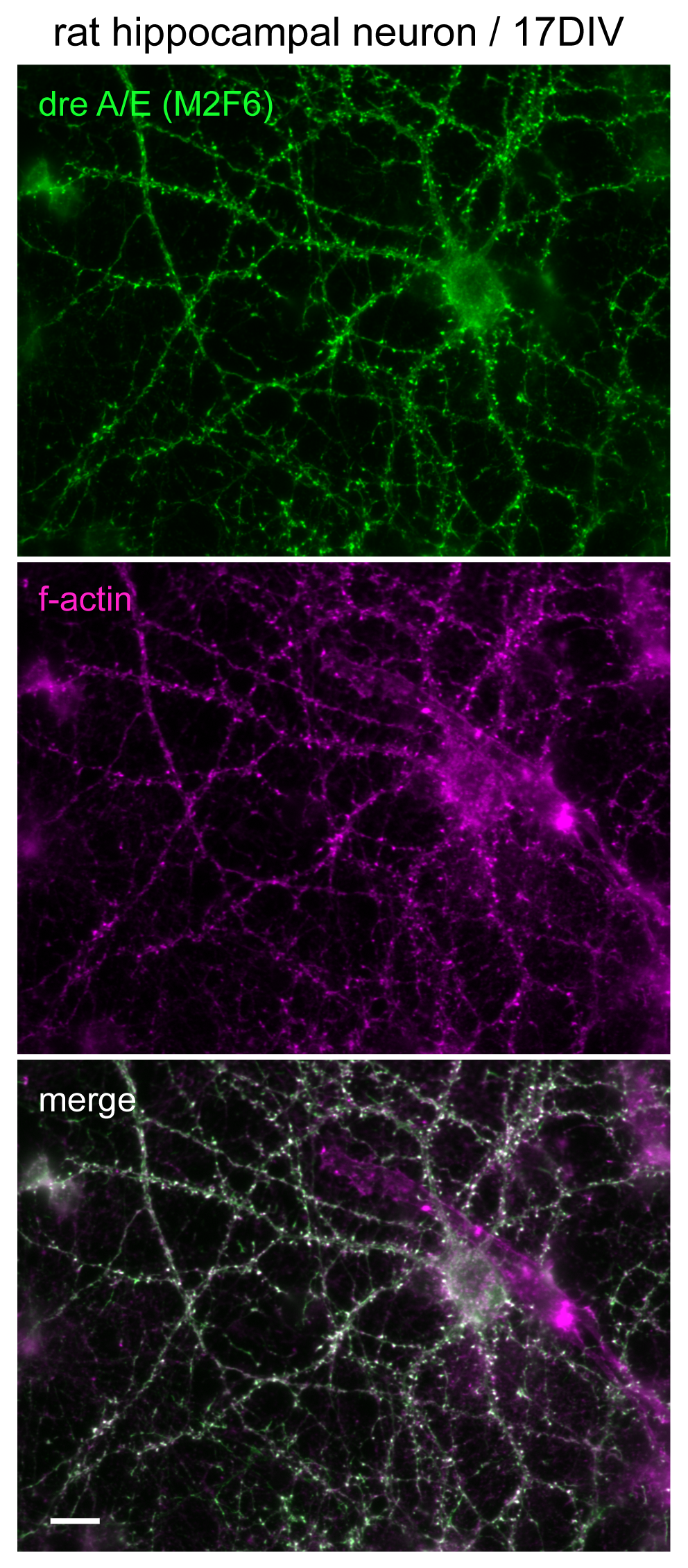

Supplement: S1 Fig — Immunostaining of rat hippocampal neurons at 17 days in vitro (DIV) with antibody against drebrin A/E (M2F6) and phalloidin (f-actin). Scale bar: 10 μm. (TIF) [file pone.0125119.s001.tif]

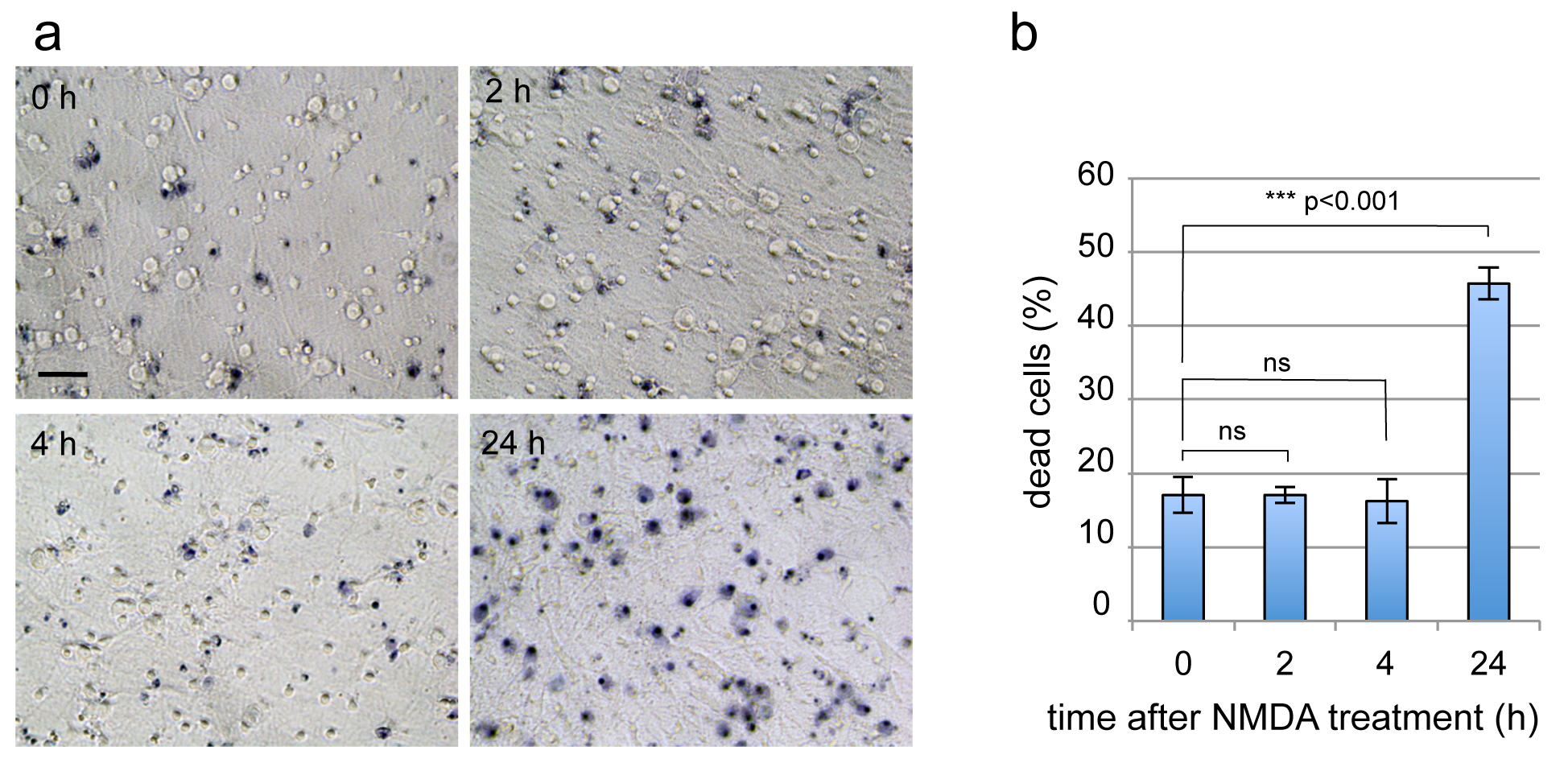

Supplement: S2 Fig — (a) Representative images of rat hippocampal neuronal cultures that were treated with NMDA for the indicated times and stained with trypan blue. Neurons were defined as cells with a large soma and extended dendrites. Scale bar: 50 μm. (b) Quantification of cell death measured by trypan blue staining. Approximately 300–500 cells were counted for each condition in each independent experiment. The numbers represent the percentages of trypan blue-positive cells. The asterisk indicates a statistically significant difference (P = 1.56 × 10^–6^ by a Tukey-Kramer test) compared with the non-treated (0 h) condition (ns, not significant). The data are represented as the mean ± standard deviation of n = 3 replicates. (TIF) [file pone.0125119.s002.tif]

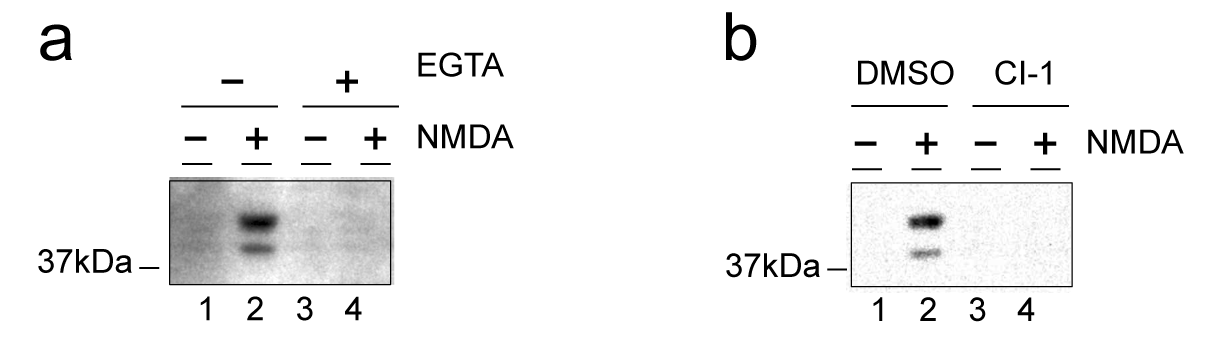

Supplement: S3 Fig — (a) Western blot analyses of drebrin A proteolytic fragments in neurons that were pretreated with EGTA for 30 min (a) or calpain inhibitor-I for 1 h (b), and then exposed to NMDA. The samples used in Fig 1C and 1E were reanalyzed using the DAS2 antibody in (a) and (b), respectively. The experiments were repeated a minimum of three times with similar results. (TIF) [file pone.0125119.s003.tif]

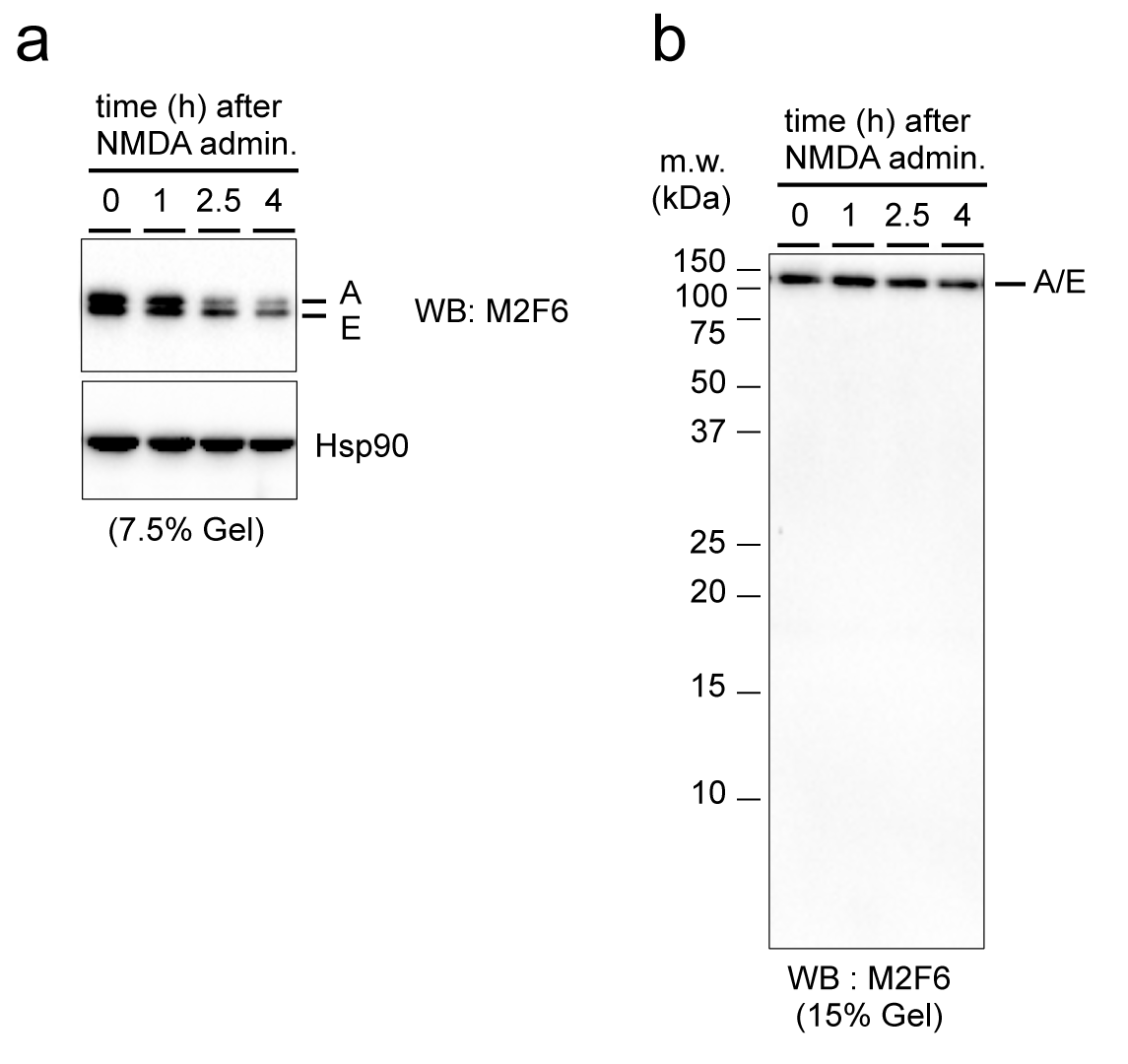

Supplement: S4 Fig — (a) Detection of NMDA-induced decreases in the levels of drebrin A and E using the M2F6 antibody. The expression level of Hsp90 was used as a loading control. (b) Lack of signals derived from degradation products recognized by the M2F6 antibody. (TIF) [file pone.0125119.s004.tif]

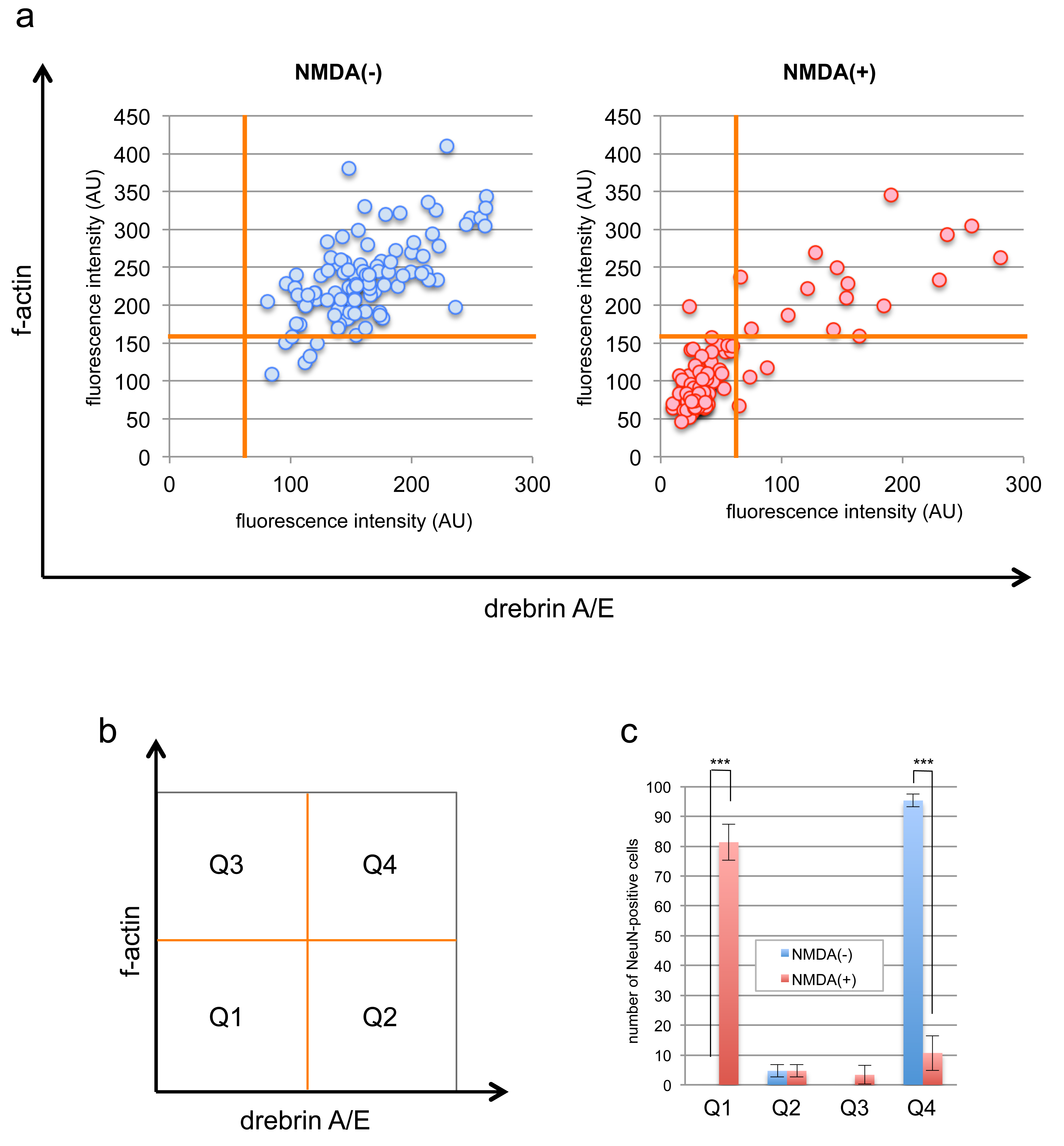

Supplement: S5 Fig — (a) The signal intensities (AU, arbitrary units) of drebrins A and E (drebrin A/E) and f-actin in circular areas (40 μm diameter) that covered the cell soma and proximal dendrites of 100 randomly selected NeuN-positive neurons. Each dot represents one neuron. The thresholds indicated by orange lines are set at 40 percent or 70 percent of median values of drebrin or f-actin in NMDA(-) condition, respectively. (b) Schematic representation showing the identification of four quadrants (Q1-Q4). (c) Statistical analysis of the numbers of neurons included in each quadrant (Q1-Q4). The data are represented as the mean ± standard deviation of n = 3 replicates. ***P < 0.005 by a Student’s t-test. (TIF) [file pone.0125119.s005.tif]

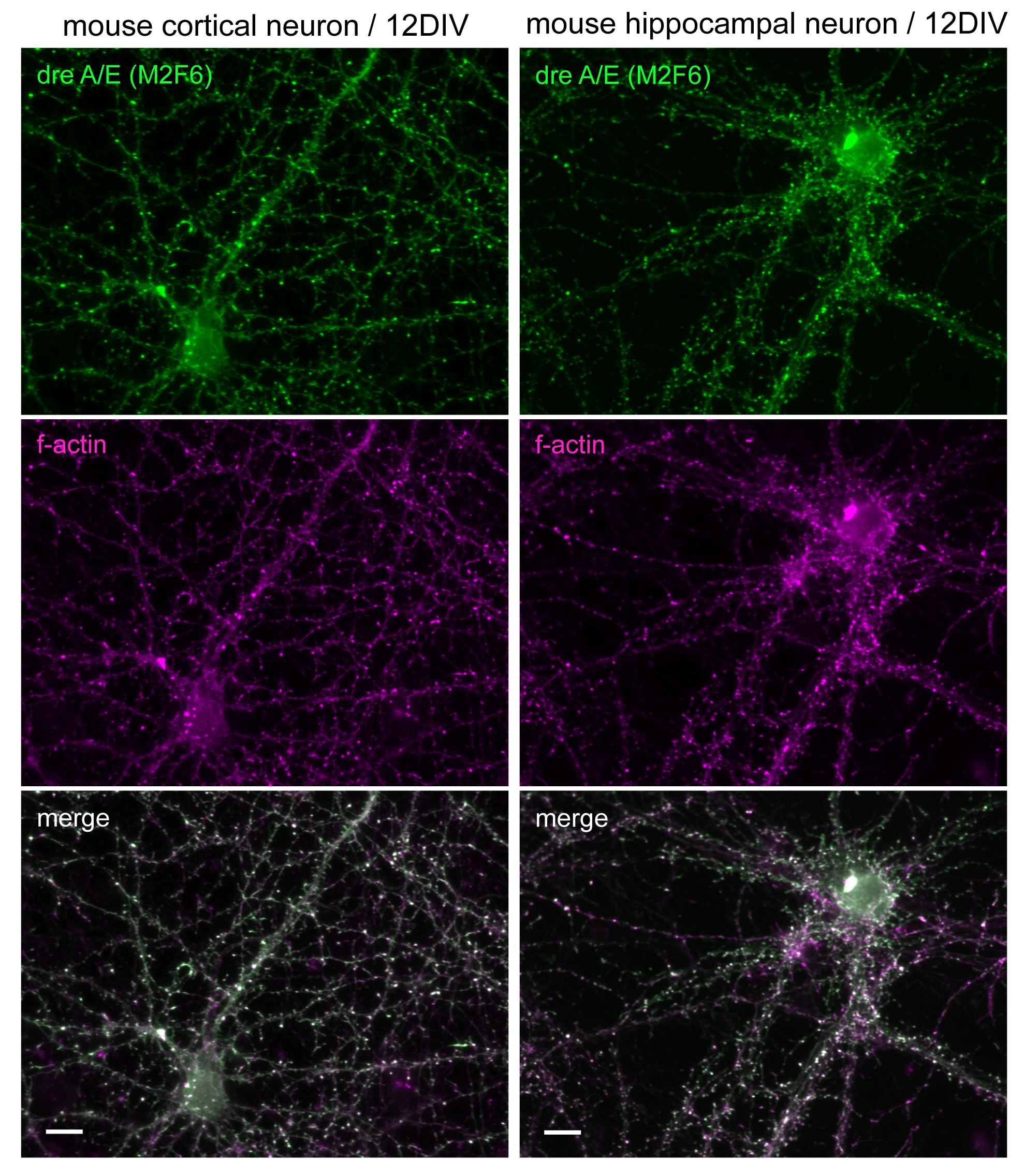

Supplement: S6 Fig — Immunostaining of mouse cortical (left panels) and hippocampal neurons (right panels) at 12 days in vitro (DIV) using antibodies against drebrin A/E (M2F6) and phalloidin (f-actin). Scale bar: 10 μm. (TIF) [file pone.0125119.s006.tif]

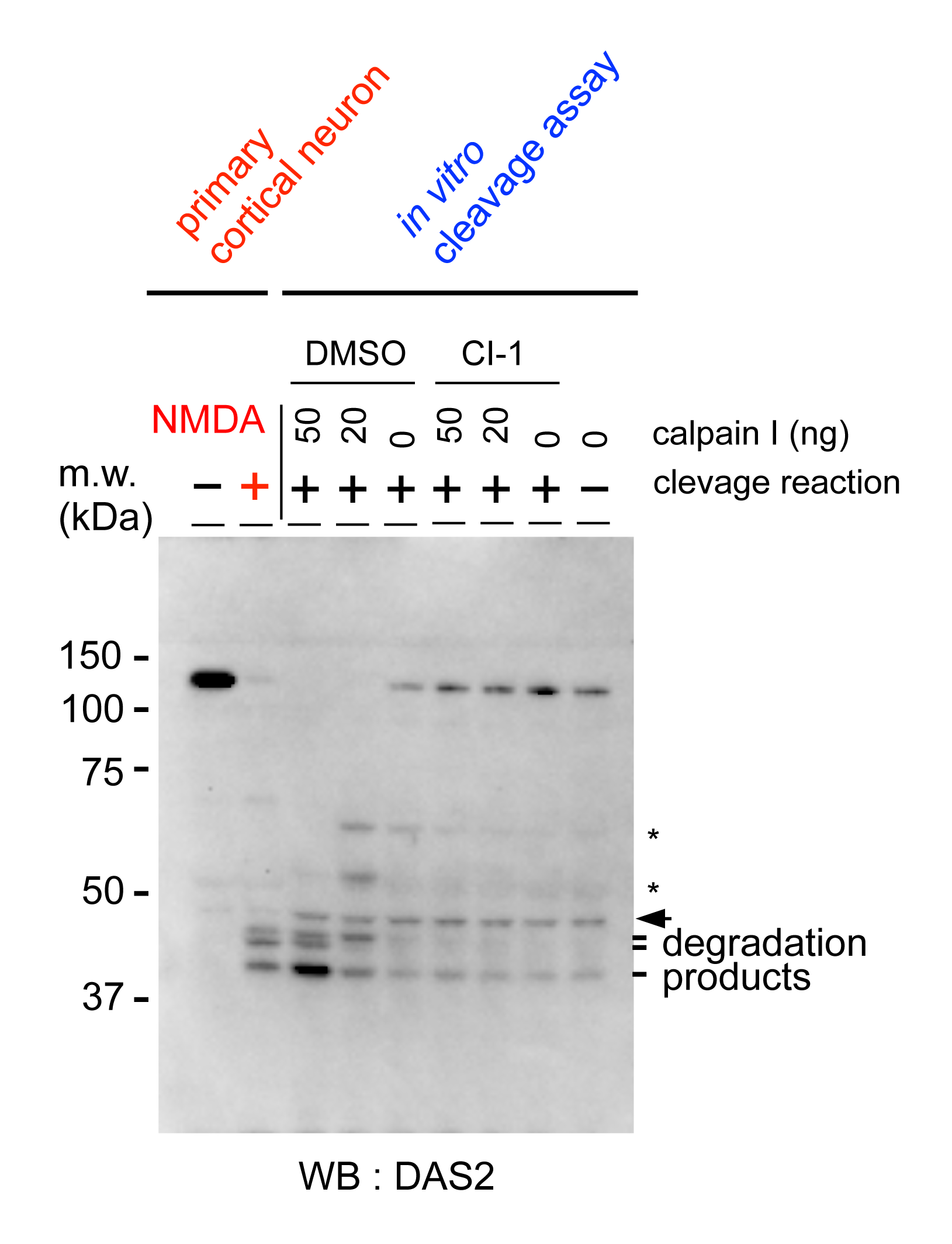

Supplement: S7 Fig — An in vitro cleavage assay using crude brain cortical extract as a substrate in the absence or presence of 100μM calpain inhibitor-1 (CI-1). The asterisks indicate the degradation products detected specifically in the in vitro cleavage assay. The arrow indicates a non-specific band. (TIF) [file pone.0125119.s007.tif]
